# Helium ion microscope – secondary ion mass spectrometry for geological materials

**DOI:** 10.3762/bjnano.11.133

**Published:** 2020-10-02

**Authors:** Matthew R Ball, Richard J M Taylor, Joshua F Einsle, Fouzia Khanom, Christelle Guillermier, Richard J Harrison

**Affiliations:** 1 Department of Earth Sciences, University of Cambridge, UK; 2 Carl Zeiss Microscopy Ltd, Cambourne, Cambridgeshire, UK; 3 School of Geographical and Earth Sciences, University of Glasgow, UK; 4 Carl Zeiss SMT Inc., Peabody, MA, USA

**Keywords:** geoscience, helium ion microscopy (HIM), lithium, secondary ion mass spectrometry (SIMS)

## Abstract

The helium ion microscope (HIM) is a focussed ion beam instrument with unprecedented spatial resolution for secondary electron imaging but has traditionally lacked microanalytical capabilities. With the addition of the secondary ion mass spectrometry (SIMS) attachment, the capabilities of the instrument have expanded to microanalysis of isotopes from Li up to hundreds of atomic mass units, effectively opening up the analysis of all natural and geological systems. However, the instrument has thus far been underutilised by the geosciences community, due in no small part to a lack of a thorough understanding of the quantitative capabilities of the instrument. Li represents an ideal element for an exploration of the instrument as a tool for geological samples, due to its importance for economic geology and a green economy, and the difficult nature of observing Li with traditional microanalytical techniques. Also Li represents a “best-case” scenario for isotopic measurements. Here we present details of sample preparation, instrument sensitivity, theoretical, and measured detection limits for both elemental and isotopic analysis as well as practicalities for geological sample analyses of Li alongside a discussion of potential geological use cases of the HIM–SIMS instrument.

## Introduction

The helium ion microscope (HIM) is a focussed ion beam (FIB) instrument, which uses a gas field ion source (GFIS) to create highly focussed beams of noble gas ions, utilising the same working principle as the field ion microscope (FIM). This was originally used to form a primary helium beam [1], but the principle of the GFIS has since been extended to the heavier noble gas neon [2] and may be applicable for even heavier noble gases such as argon. Whilst the HIM was shown to achieve exceptional imaging resolution using secondary electrons generated by the primary ion beam [3–6], it lacked microanalytical capabilities. There were several suggestions for the possibility of microanalysis on the HIM, the most common of these being Rutherford backscattered ion imaging (RBI) and secondary ion mass spectrometry (SIMS) [7–9]. However, the variation of RBI intensity with changing surface chemistry, specifically the atomic number, *Z*, reaches a plateau at relatively low *Z* values of around 50 [7], limiting its use to lighter elements only. SIMS, however, opens the entire range of mass numbers, from a few atomic mass units up to several hundred atomic mass units, whilst also leaving open the possibility of in situ isotopic measurements. This combined HIM–SIMS instrument has intriguing possibilities for geological materials as, unlike previous SIMS techniques limited by the probe size of the primary beam, the small beam size theoretically allows for chemical mapping at high sensitivity with the spatial resolution controlled only by beam–sample interactions [9]. The possibility of isotopic measurements at such a high spatial resolution, around 10 nm, is of particular importance in the planetary sciences, where variations are extreme in both their magnitude and their nanometre-scale size [10–11]. Additionally, planetary materials pose a particular challenge to traditional microanalysis techniques, since they are often rare or one-of-a-kind samples, limiting them to non-destructive analysis, or analysis that preserves as much of the sample as possible. They also typically contain very small components, which record the signals of different stellar processes [10,12], resulting in extreme heterogeneity of data across nanometre-scale distances. Ion imaging in cosmochemistry is typically performed with the NanoSIMS instrument, which can reach spatial resolutions of 50–100 nm for Cs^+^ with a beam current below 1 pA and 200–400 nm for O^−^ with a beam current below 10 pA [13]. However, the scale of some inclusions within some planetary materials can be of the order of hundreds of nanometres, making detailed imaging of such inclusions with the NanoSIMS unfeasible. The HIM–SIMS however, with spatial resolutions of less than 10 nm, represents an ideal tool for such imaging, allowing for detailed nanometre-scale chemical imaging over large areas.

Whilst many geological materials could benefit from the analytical capabilities of the HIM–SIMS, they also present challenges for the device. In particular, the insulating nature of silicates, which make up most geological materials on Earth, requires additional sample preparation, which, if performed incorrectly, can have a negative impact on both qualitative and quantitative use of the instrument. Here, we demonstrate the instrumental sensitivity, capability, and repeatability of the HIM–SIMS using Li as a test element. The choice of Li is particularly relevant for geoscience applications as it represents a key geological resource for green energy storage, a challenge to the commonly used scanning electron microscope (SEM)-based microanalysis methods, which rely on energy/wavelength dispersive X-ray spectroscopy, as well as a best-case scenario for isotope measurements, since it has one of the largest relative mass differences between isotopes of any element. Alongside this, the practicalities of sample preparation and a discussion of further potential use cases of the HIM–SIMS for geological materials are also provided.

## Results and Discussion

### Methods

All analyses were performed using an ORION NanoFab HIM with an attached V500 double focussing magnetic sector mass spectrometer [14]. The gas field ion source (GFIS) of the ORION instrument produces a highly focussed single ion stream of He^+^ or Ne^+^, with a very small probe size (0.5 nm for He^+^ and less than 3 nm for Ne^+^). Primary ion currents of 1–10 pA were applied to the samples with acceleration voltages between 10,000 and 20,000 keV. Positive secondary ions of Li^+^ were extracted by the application of a 500 V extraction voltage applied to the sample surface along an extraction system mounted directly above the sample, which subtends a large solid angle at the sample for a high collection efficiency. Ions were then measured by electron multiplier detectors set to fixed positions at *m*/*z* 6, *m*/*z* 7, and an arbitrary value of *m*/*z* for which no secondary ions were expected, for the measurement of a “background count rate”, with a fixed, low magnetic field of around 100 mT. The primary beam was rastered over the sample to simultaneously map ion counts on each detector with a typical dwell time per pixel of 4 ms, leading to an average mapping time of 20 min.

### Sample preparation

#### Sample mounting

Unlike many traditional SIMS instruments, the HIM–SIMS has fewer constraints on sample mount size, as the sample holder is unmodified from the holder of the HIM, allowing for larger mounts, with space for multiple thin sections alongside standards. Whilst a large sample holder allows for multiple samples at a time, with fewer concerns on mount size, care must be taken to maintain a constant height across the mounts, as the extractor sits at a constant height above the holder for a given focus, which can lead to potential collisions with other samples if the heights and, therefore, focal distance is not constant.

#### Sample coating

**Figure 1:**
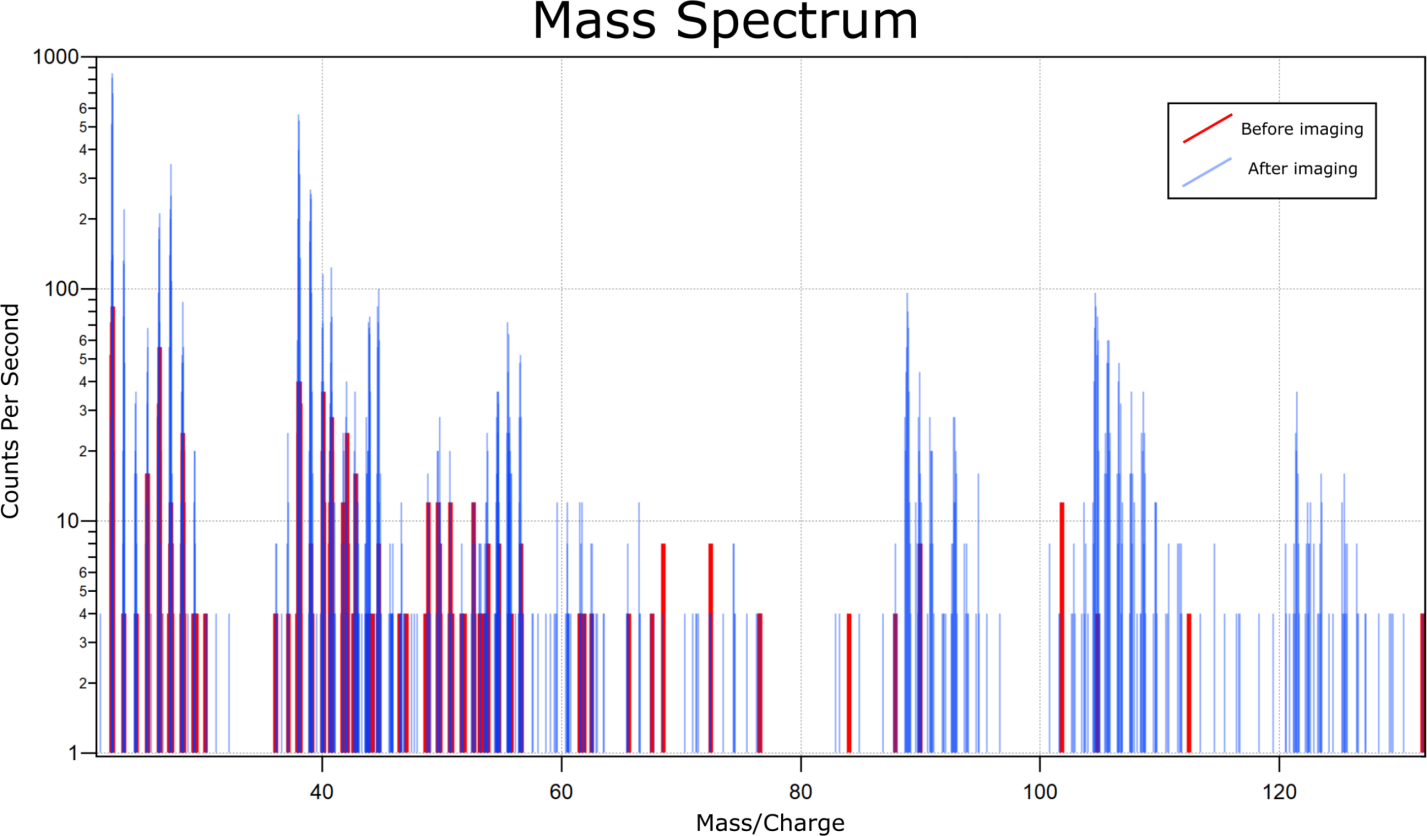
Mass spectra of a natural zircon sample before rastering with the primary beam (red) and after rastering with the primary beam for 240 min (blue). Count rates increase dramatically after the sample coating has been removed.

**Figure 2:**
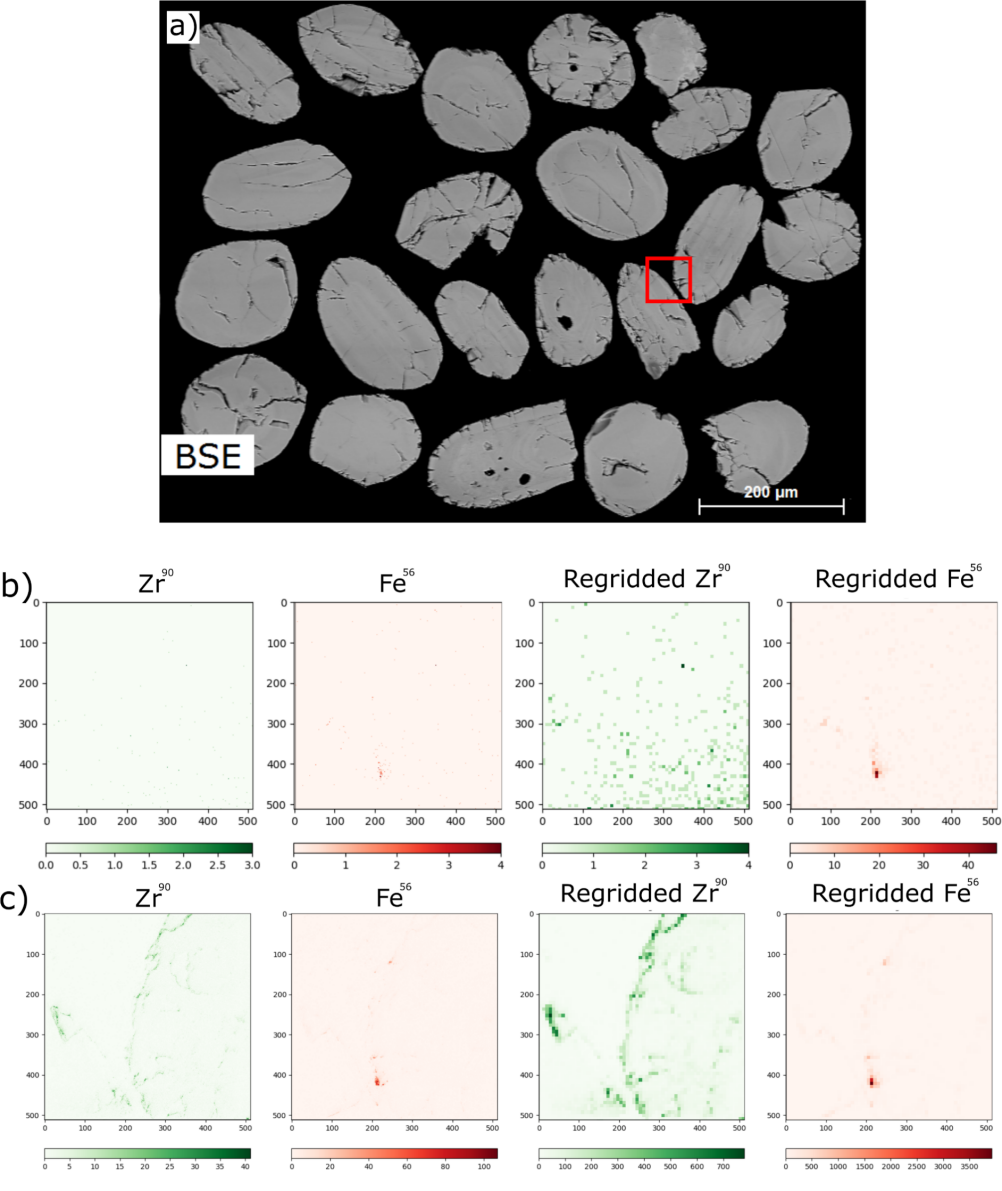
a) Backscattered electron (BSE) map of the sample, SIMS location outlined in red; b) raw and processed ^90^Zr and ^56^Fe mass images after 20 min of rastered beam exposure on the sample; c) raw and processed ^90^Zr and ^56^Fe mass images after 240 min of rastered beam exposure on the sample. Initial maps were 512 × 512 pixels, covering a field of view of 70 µm. The scales show the absolute number of ions detected.

Due to size constraints within the chamber, it is currently not possible to have both an electron flood gun and SIMS attachment on the same device. This is a problem for geological samples, as the vast majority of them are insulators. To counteract this insulating behaviour and prevent the buildup of charge on the sample surface, samples must be coated with an electrically conducting material before they are introduced to the instrument. Traditional SIMS instruments apply a thin metallic coating, typically gold, using a sputter coater. Here we employ a similar technique, coating samples with either gold or platinum, or, in the case of analysis of platinum-group elements [15], carbon. This coating is removed by the action of the primary beam in the region of interest prior to analysis, allowing for the removal of charge at the edges, but exposure of the sample surface within the analytical area. Figure 1 shows a mass spectrum taken on a natural zircon grain from NW Scotland, UK, mounted in epoxy resin with a carbon coating. Count rates before rastering with the primary beam are shown in red, whilst count rates from the same sample area are shown in blue, after rastering with the neon beam to remove the carbon coating. The count rate increases dramatically after the removal of the carbon coating. For some mass/charge values, which were not present in the original mass spectrum, such as the ZrO^+^ fragment at *m*/*z* 106 and the ZrO_2_^+^ fragment at *m*/*z* 122, signals became measurable significantly above the background noise. Figure 2 shows the same zircon sample as Figure 1, immediately after the first mass spectrum in Figure 2b and immediately before the second mass spectrum in Figure 2c. The dramatic increase in signal is again demonstrated here, with almost no signal from the ^90^Zr^+^ in Figure 2b, in contrast to the same mass-filtered image in Figure 2c. This demonstrates how important the removal of the conductive coating is for analysis of unknown material. While the coating is necessary for conductivity across the sample surface, it can dampen the signal to a point where some elements fall below the background noise level.

However, the primary beam is not designed for the rapid removal of material and if the applied coating is thicker than 5–10 nm, its removal can take an unreasonable amount of time. The increase in signal shown in Figure 1 and Figure 2 comes after 240 min of rastering with the primary neon beam, which corresponds to almost 300 min of instrument use, an unfeasible time for a single image. One solution is to pre-raster the areas of interest with a gallium beam before the sample is taken to this instrument, but this leads to the implantation of gallium ions and can lead to potential damage to the sample if an incorrect dose is used. Instead, care should be taken to apply thin (5–10 nm) coating layers to the sample, to maximise analysis time on the instrument.

#### Sample polishing

Figure 2 also demonstrates another issue with sample preparation, that is, polishing. Polishing is critical for the extraction of quantitative information using the SIMS method, since surface relief distorts the extraction field, enhancing extraction of secondary ions at topographic highs relative to lows, distorting the distribution of counts from the true distribution within the sample. The intensity variation is further modified by variations in the sputter yield at areas with a grazing incidence angle. This can be seen in Figure 2. Most of the signal comes from the edges of the zircon grains, where the zircon is raised relative to the epoxy. The formation of relief is a balancing act for SIMS analysis. During HIM–SIMS all the typical effects are exaggerated, therefore requiring an exceptional polish as well as minimal relief. However, whilst techniques exist to reduce relief to a minimum, for example, lapping films, they may not yield a polish smooth as lapping cloths do. As a result, it is almost impossible to satisfy both requirements. The recommendation should be to minimize relief as much as possible using appropriate preparation techniques for the sample before a final polish (here we ended with a 0.25 µm polish). However, even after minimising the effect of relief, samples should be analysed away from the sample edge in order to obtain quantitative data.

### Quantification

#### Concentration calibration

Quantification of concentrations using SIMS data is typically performed using one of two approaches. These are the useful yield approach [9] and the relative sensitivity factor approach [16], both of which rely on the effective use of appropriate standards. The useful yield approach depends on the calculation of the useful yield of an element, defined as the ratio of detected ions of species x to the number of sputtered atoms of x, in a matrix-matched reference sample. The concentration of x, C_x_, can then be calculated using Equation 1, and depends on the secondary ion current, *I*_x_, the calculated useful yield UY_x_, the primary ion beam current, *I*_p_ and the sputtering yield, *Y*, which is typically measured after the analysis:

[1] $$C_{\text{x}}=\frac{I_{\text{x}}}{{\text{UY}}_{\text{x}}\cdot I_{\text{p}}\cdot Y}.$$

**Figure 3:**
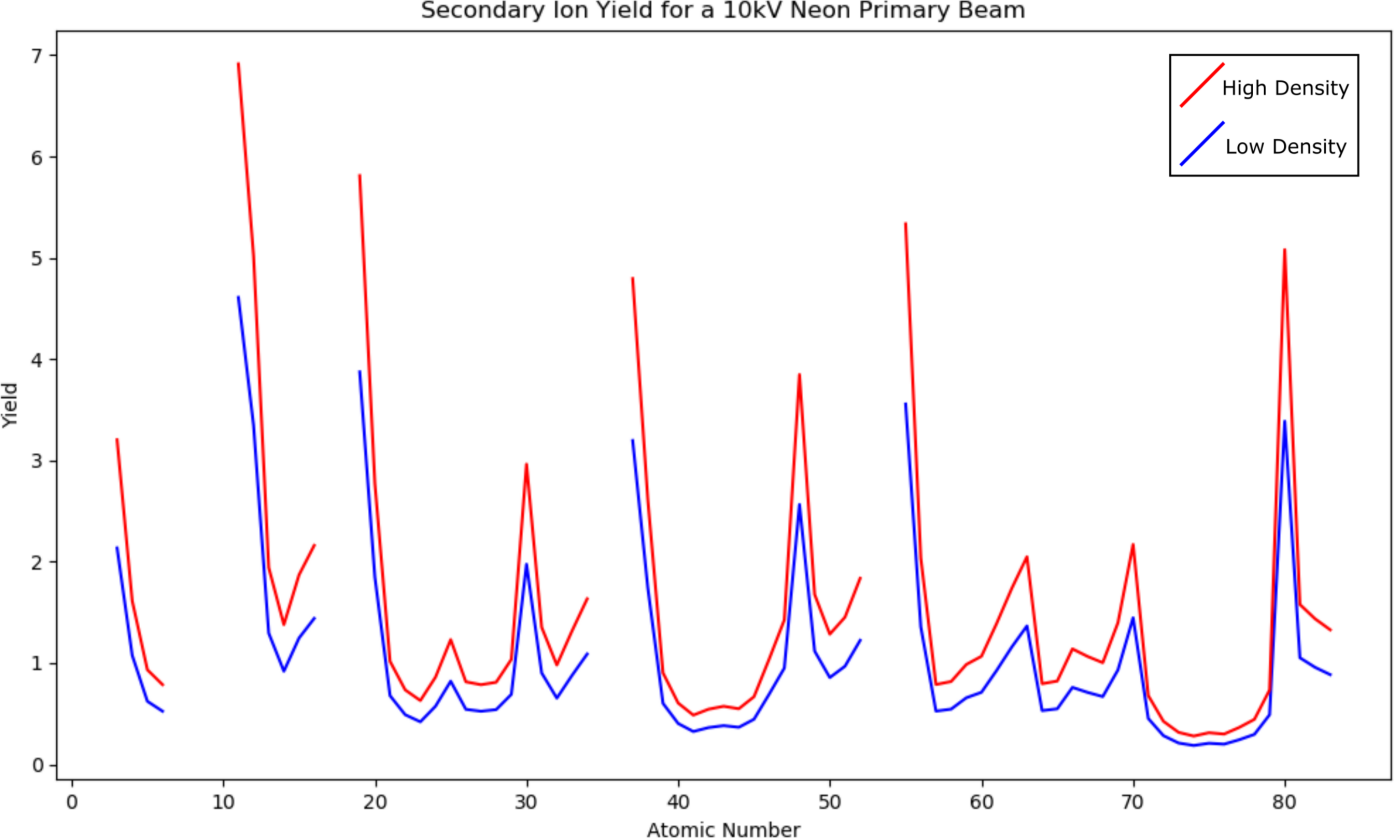
The calculated sputtering yield as a function of the atomic number for a 10 kV primary Ne beam impacting a silicate glass matrix for a low-density glass (2.2 g·cm^−3^, blue) and a high-density glass (3.3 g·cm^−3^, red), calculation after [17].

The sputtering yield depends on the nature of the atoms as well as the matrix in which they are bound. This yield can be predicted semi-empirically for a primary Ne beam [17]. Figure 3 shows how the calculated yield of different atoms varies for a silicate glass matrix with two different densities, 2.2 and 3.3 g·cm^−3^, that is, respectively, low-density and high-density silica glass. Different matrix materials can also affect the ionization probability of the sputtered material. Thus, it is necessary to properly match the matrix of the samples and standards, since small changes in the matrix can have a large effect on the constants involved in calculating the concentration of an element.

The relative sensitivity factor approach relies on the measurement of both the target element, x, as well as a known matrix element, m. The concentration of the element x, *C*_x_, can then be calculated using the secondary ion current of species x, *I*_x_, the secondary ion current of the matrix element m, *I*_m_, and the relative sensitivity of the two elements, which is calculated using a matrix-matched standard under the same beam conditions. This approach can be simplified by relating the concentration of an element within a standard to the secondary ion current for that element from the standard and applying this correction to the secondary ion current measured on a sample under identical beam conditions. This simplified approach does not account for variations in measurement conditions, which are accounted for in a “true” relative sensitivity factor approach by the secondary ion current of a matrix element, *I*_m_, as shown in Equation 2:

[2] $$C_{\text{x}}=\frac{I_{\text{x}}}{I_{\text{m}}}\cdot {\text{RSF}}_{\text{x}}.$$

Both of these methods rely on calibration to a known standard and using standards on the HIM–SIMS comes with many of the same necessities as on a traditional SIMS instrument, such as matrix-matching samples and standards. However, an additional problem when using standards on the HIM–SIMS is the homogeneity of the standard material. Whilst an electron microprobe standard should be homogeneous on a length scale of several micrometres, this same standard is required to maintain this homogeneity at a nanometre length scale in order to be used accurately with the HIM–SIMS instrument. It is extremely difficult to manufacture a standard material for all elements that is homogeneous at the nanometre scale. Platinum group elements, for example, commonly form micrometre-scale nuggets [18–19].

#### Isotopic calibration

Isotopic quantification also relies on the use of matrix-matched standards that are homogeneous on the nanometre length scale. Measured isotopic variations are typically expressed in delta notation. An example calculation for Li isotopes is shown in Equation 3. This accounts for the small variations in raw isotopic ratios typically seen in geological materials.

[3] $$\delta^{7}\text{Li}=\left\{\frac{\left({\left[\frac{{}_{}^{7}\text{Li}}{{}_{}^{6}\text{Li}}\right]}_{\text{sample}}-{\left[\frac{{}_{}^{7}\text{Li}}{{}_{}^{6}\text{Li}}\right]}_{\text{standard}}\right)}{{\left[\frac{{}_{}^{7}\text{Li}}{{}_{}^{6}\text{Li}}\right]}_{\text{standard}}}\times 1000\right\}$$

#### Data processing

Due to the small size of the primary ion beam interaction of less than 10 nm [9,20], combined with typical primary ion beam currents in the range of 10–100 pA, a very small volume of material is removed during each interaction between beam and sample surface. Whilst this makes the instrument extremely surface sensitive, it can also result in very low count rates for low concentrations and/or low-yield elements. However, even in such cases, appropriate data processing techniques can extract data with meaningful counting statistics for elemental and isotopic analysis, although this processing does have consequences, for example, it can reduce the lateral resolution significantly.

**Figure 4:**
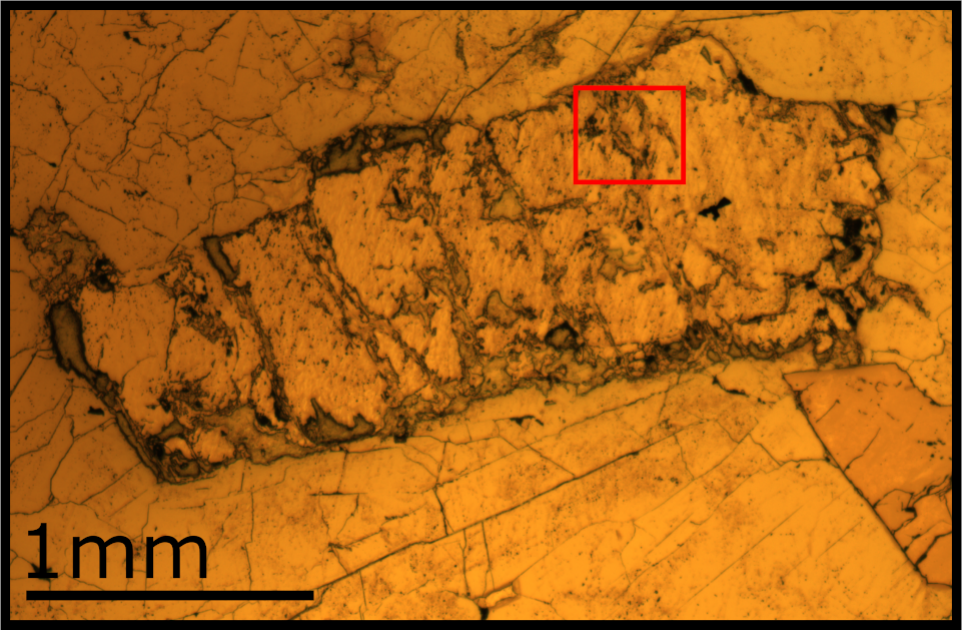
Reflected-light micrograph of the analysed Spodumene grain. The grain has relatively unaltered regions, separated by altered regions which intrude from the edge of the grain. Figure 5 was taken from within the region shown in red, across one such zone of alteration.

**Figure 5:**
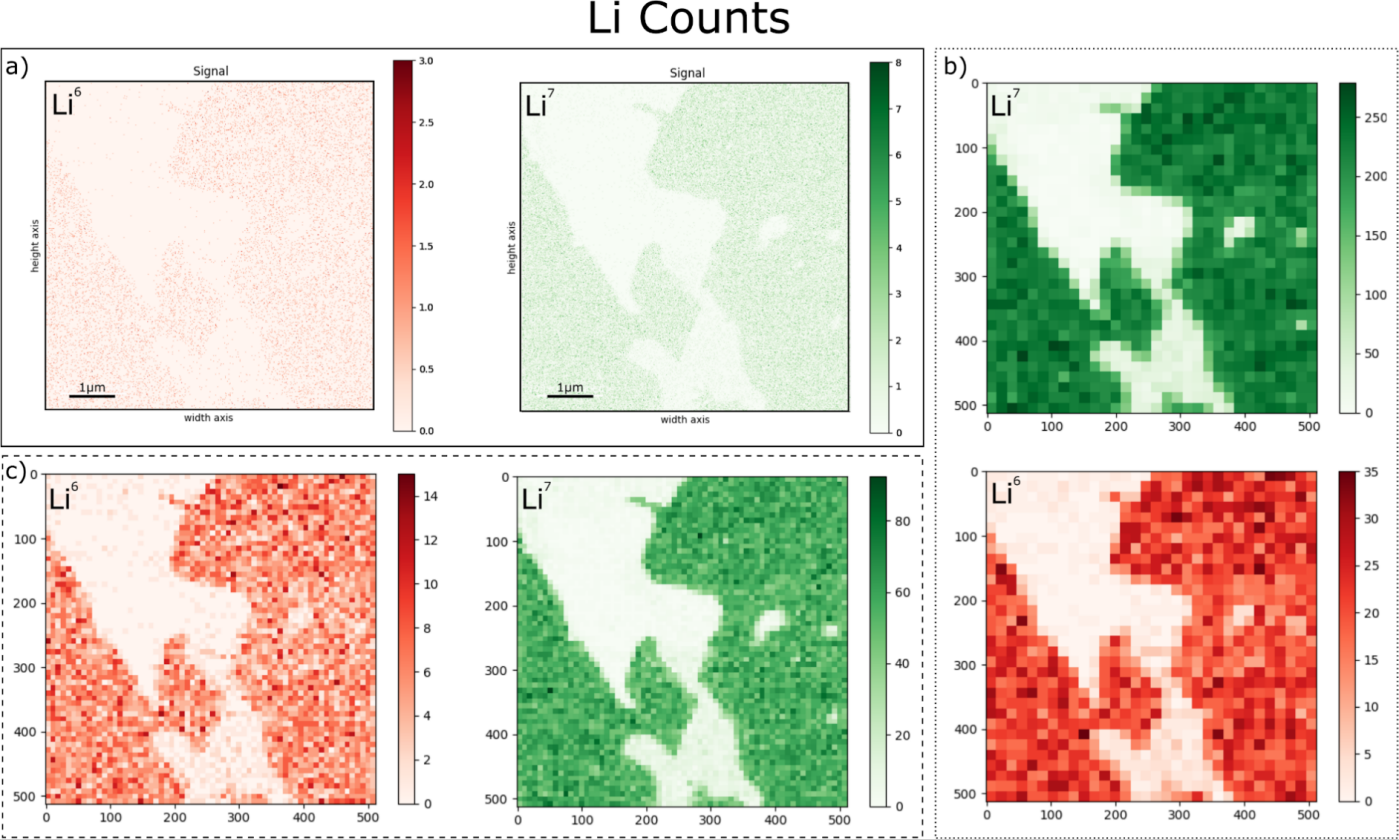
a) Raw (solid line), b) 32 × 32 regridded (dotted line) and c) 64 × 64 regridded (dashed line) maps of ^6^Li (red) and ^7^Li (green) from a Spodumene grain, see Figure 4.

**Figure 6:**
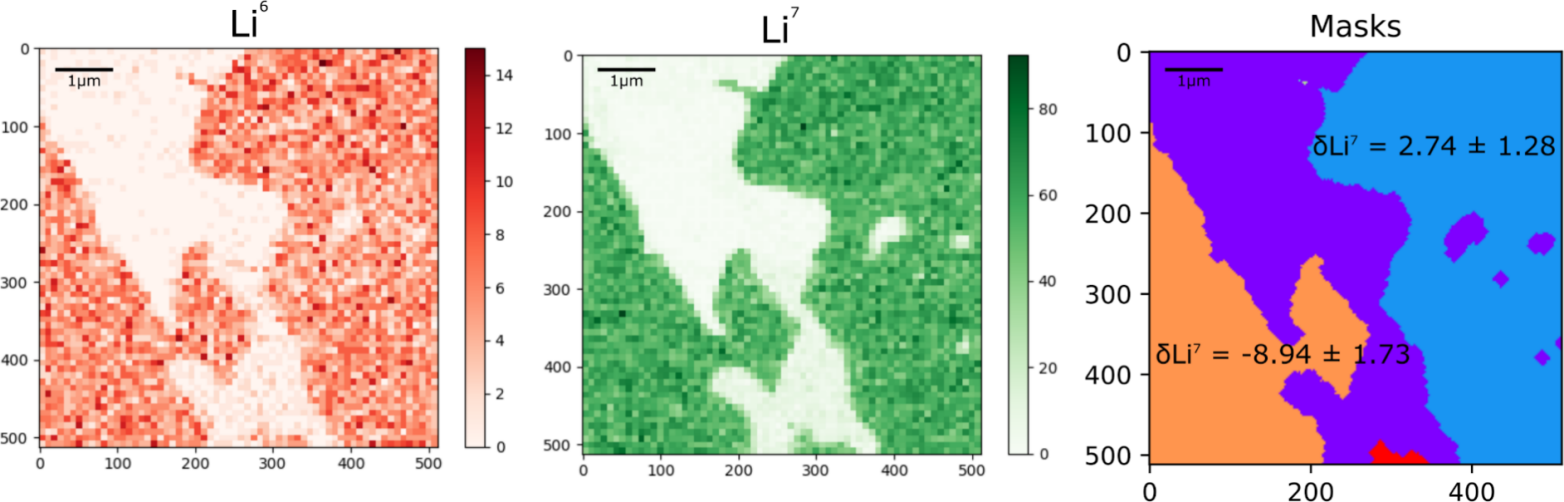
The relative δ^7^Li ratios for regions within the same grain of Spodumene as in Figure 4 and Figure 5. Four connected regions with roughly similar counts have been identified, the purple region, an altered region, shows no Li, whilst the red region contains too few pixels to calculate a δ^7^Li value with a reasonable error. Values are not calibrated to an external standard, but instead to the average ratio across all measurements of the grain, which should lead to values of zero for isotopically homogenous material.

One method for increasing count rates is to regrid data, effectively reducing pixel number and image resolution whilst increasing pixel size and counts per pixel. Figure 5 shows ^7^Li and ^6^Li maps taken from a thin section containing the economic Li-bearing mineral Spodumene (LiAlSi_2_O_6_) as raw and regridded data, with different regridding sizes, taken across a region of alteration. The raw maps in Figure 5a can be used to define the altered region from the unaltered regions. Within the unaltered region are pixels with zero counts for either isotope of Li. Since we can assume variations in Li concentration are not occurring on a wavelength similar to the pixel size (14.6 nm), these must be measurement error. The regridded maps act to remove these anomalous pixels. The inverse relationship between counts per pixel and resolution can be clearly seen. However, in some respects regridding the data in this manner reduces the advantages of the extreme spatial resolution offered by HIM–SIMS. Another method is to sum counts over individual regions of the data, maintaining the lateral resolution of the data, but increasing the count rates under the assumption that different regions have roughly homogenous concentrations of an element or isotope ratios. In the case of the data shown in Figure 6, the regions can be separated into Li-bearing Spodumene and Li-free regions of altered Spodumene, which intrude from the edges of the grain, as shown in Figure 4. These Li-bearing regions can be further separated on the basis of connectivity. The ^7^Li and ^6^Li data can then be used together to calculate the variability of isotopic ratios across the region of alteration, as shown in Figure 6, where the shape and outline of the regions can be seen in comparison to the regridded data with lower spatial resolution.

Irregular regridding can also be used to create effective line profiles along features that are at least as wide as the field of view of one HIM–SIMS map. This data can then be regridded to sum along an axis perpendicular to the beam rastering direction, which should negate any effects of variable beam conditions. This is particularly useful for isotopic analysis, as it generates data with significantly better internal error/counting statistics [21], whilst still summing data from a width that is much smaller than a traditional SIMS instrument line profile.

Another method to increase counts is to image the same area sequentially. This method has been used for 3D imaging of extremely thin materials. However, for most geological samples the thickness of any layer of interest is much greater than the approx. 10 nm of removed material per map. In this way multiple maps may be summed, on the assumption that the region of interest is thicker than the total removed material [15]. This method is effective only when the surrounding area remains able to compensate the charge and the electric field is not so distorted as to prevent the removal of the generated secondary ions.

### Geological applications

#### Light elements

Some of the most important elements in the geosciences have low atomic numbers. Lithium, for example, is critical to a low-carbon energy landscape through electric vehicles and battery materials [22–23]. The global carbon cycle is likewise critical for the continued evolution of surface conditions on Earth, both in the geological record [24–25] and for the future [26]. Oxygen is a critical element as it is both one of the main constituents of the most common geological materials, silicates, and one of the best understood isotopic systems [12,27]. However, mapping the distribution of these elements by traditional methods, typically using SEM–EDS techniques, is extremely difficult, as the characteristic X-rays produced by these elements have such low energies that they are prone to re-absorption within the sample as well as being difficult to separate from other peaks in the produced spectra. This makes quantification almost impossible. The HIM–SIMS, in contrast, has its highest mass resolving power at low masses (around 400 *M*/Δ*M*) with a low magnetic field applied within the mass spectrometer, making it an ideal tool for mapping these elements.

#### Lithium mapping

**Figure 7:**
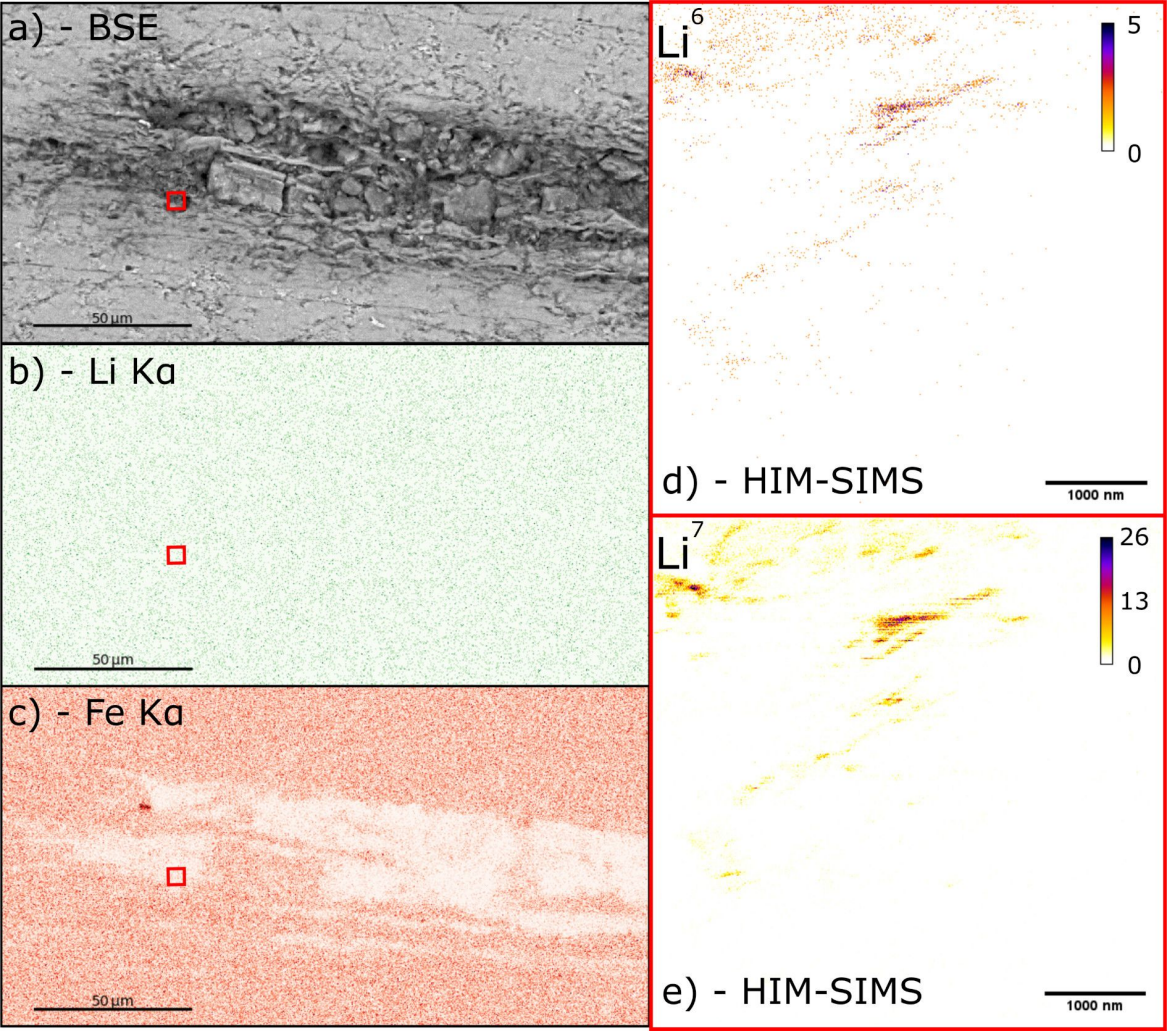
a) Backscatter electron image of the region of interest. The red square shows the region mapped using HIM–SIMS; b) Li EDS map of the region shown in a); c) Fe EDS map of the region shown in a); d) ^6^Li map of the region within the red square in a) using HIM–SIMS; e) ^7^Li map of the region within the red square in a) using HIM–SIMS.

Figure 7 shows a comparison of the SEM–EDS signal obtained from a sample of Li-bearing mica and the HIM–SIMS signal from a subregion of the same sample. Both isotopes of Li can be clearly seen in HIM–SIMS where no Li signal can be detected above background noise in the SEM–EDS data. The Fe signal is also shown, which should be inversely proportional to the Li signal as Fe and Li substitute in the crystal lattice within a solid solution. The Li signal appears to be stronger along mica sheets perpendicular to the *c*-axis of the crystal structure. However, this may be the result of surface topography similar to that observed in the zircon samples, as a result of polishing picking out the *c*-axis of the crystal structure, instead of a geological effect, such as greisenization.

**Figure 8:**
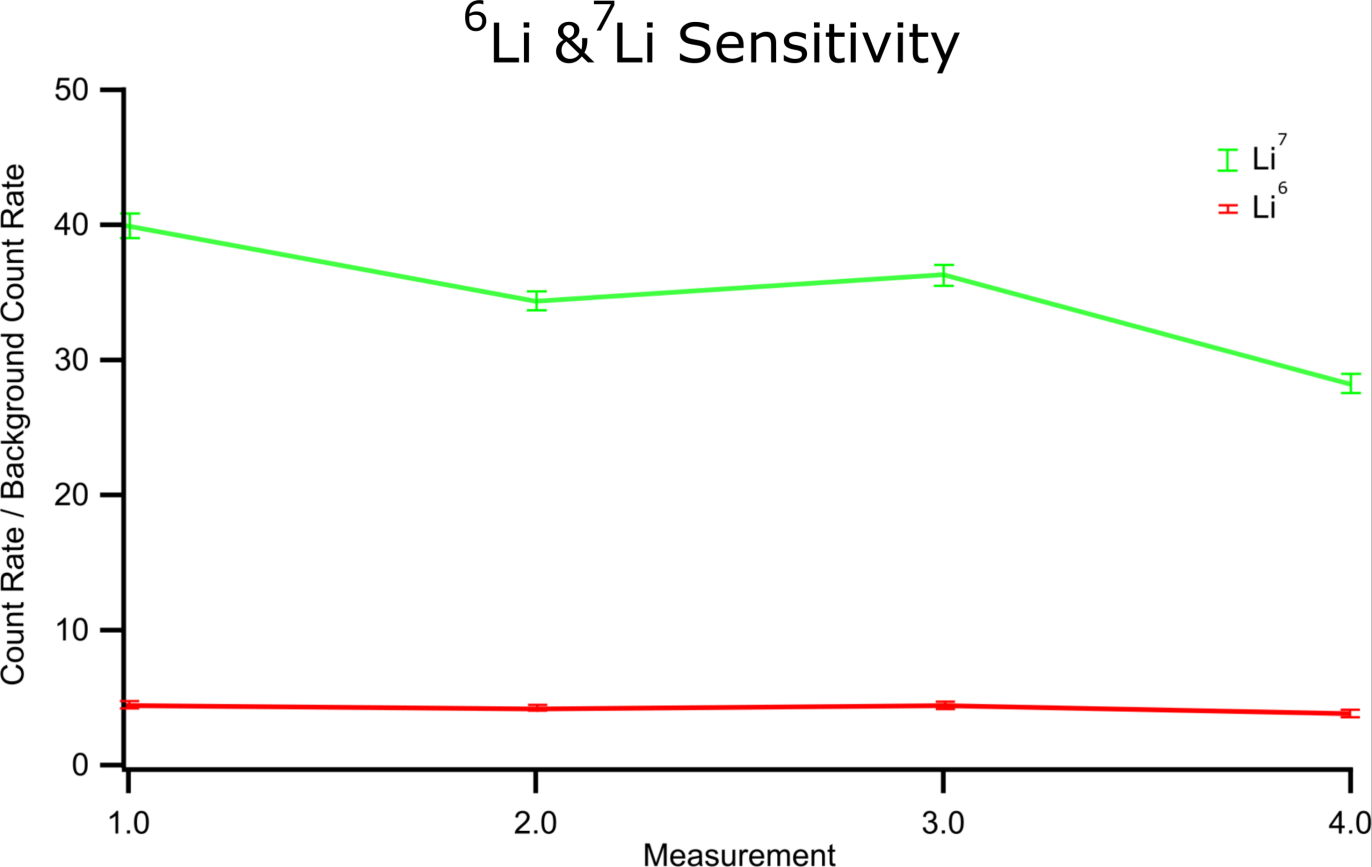
Count rates relative to background count rates for ^6^Li (red, 3 μg/g) and ^7^Li (green, 37 μg/g) for the NIST 612 glass standard. Background counts were collected simultaneously on a detector positioned over a fractional mass/charge ratio with no real counts.

The sensitivity toward Li has also been investigated in NIST glass standards. The NIST 612 standard has a known total Li concentration of 40 µg/g [28], which corresponds to concentrations of 37 µg/g for ^7^Li and 3 µg/g for ^6^Li. With one detector positioned at a fractional mass/charge ratio, which does not correspond to any elemental mass/charge ratio or half mass/charge ratio, the background count rate can be measured alongside the target mass/charge values. Figure 8 shows the count rates of ^6^Li and ^7^Li for NIST 612 glass relative to this background count rate, showing an experimentally derived instrumental sensitivity greater than 3 µg/g for Li.

#### Lithium isotopes

**Figure 9:**
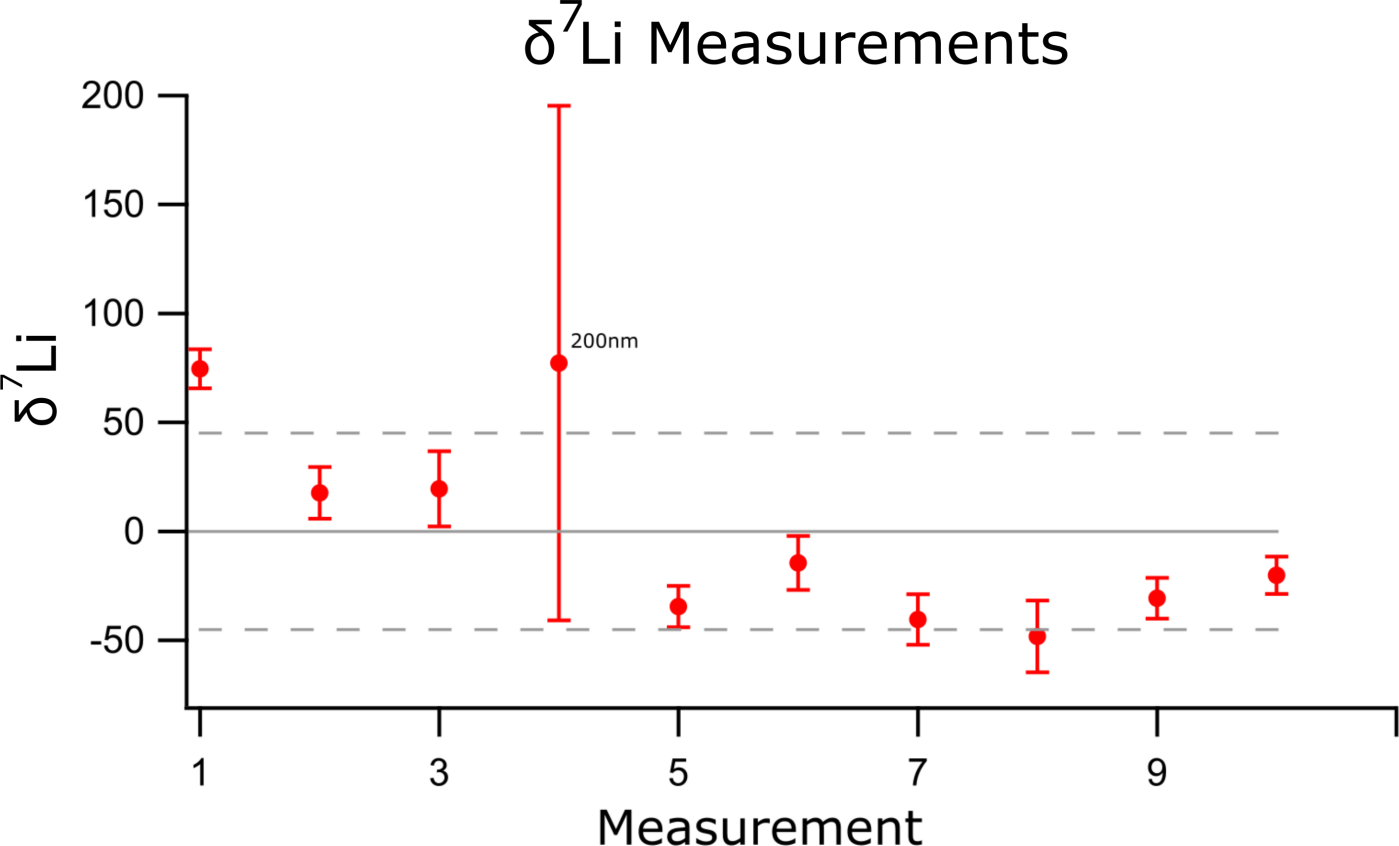
The relative δ^7^Li ratios for regions within the same grain of Spodumene. Values are not calibrated to an external standard, but instead to the average ratio across all measurements, which should lead to values of zero for isotopically homogeneous material. The internal statistical counting error is shown in red for each point with the external error across all measurements in grey.

Lithium isotopes can be used as a tracer of important geological processes [29], responding readily to fractionation due to the large percentage mass difference between the two isotopes, ^6^Li and ^7^Li. Using the Li isotope system within a single sample of unaltered spodumene, the internal error due to counting statistics [21] and the external standard error over multiple measurements has been investigated. Figure 9 shows the δ^7^Li values measured on a spodumene sample, calculated relative to the average ratio across all measurements, rather than relative to a standard as in Equation 3. Due to this, accurate measurements would always sit within the error of a δ^7^Li value of 0. All measurements were taken over an area between 10 and 30 µm, except for the fourth point, which was over a 200 nm area. For the latter measurement with a 128 × 128 pixel map, the pixel size (1.5 nm) is smaller than the interaction volume of the beam, reducing the count rate significantly. The first measured pixel sputters material from a region effecting many more pixels, reducing any signal from these pixels when the beam does “measure” these points. Whilst this does drastically increase the internal error of this measurement, the data is still within the error of 0. The external error (ca. ±48‰) also appears to be negatively affected by this 200 nm data point, but is not significantly lower without this point included, remaining higher than the terrestrial range of variation [30].

**Figure 10:**
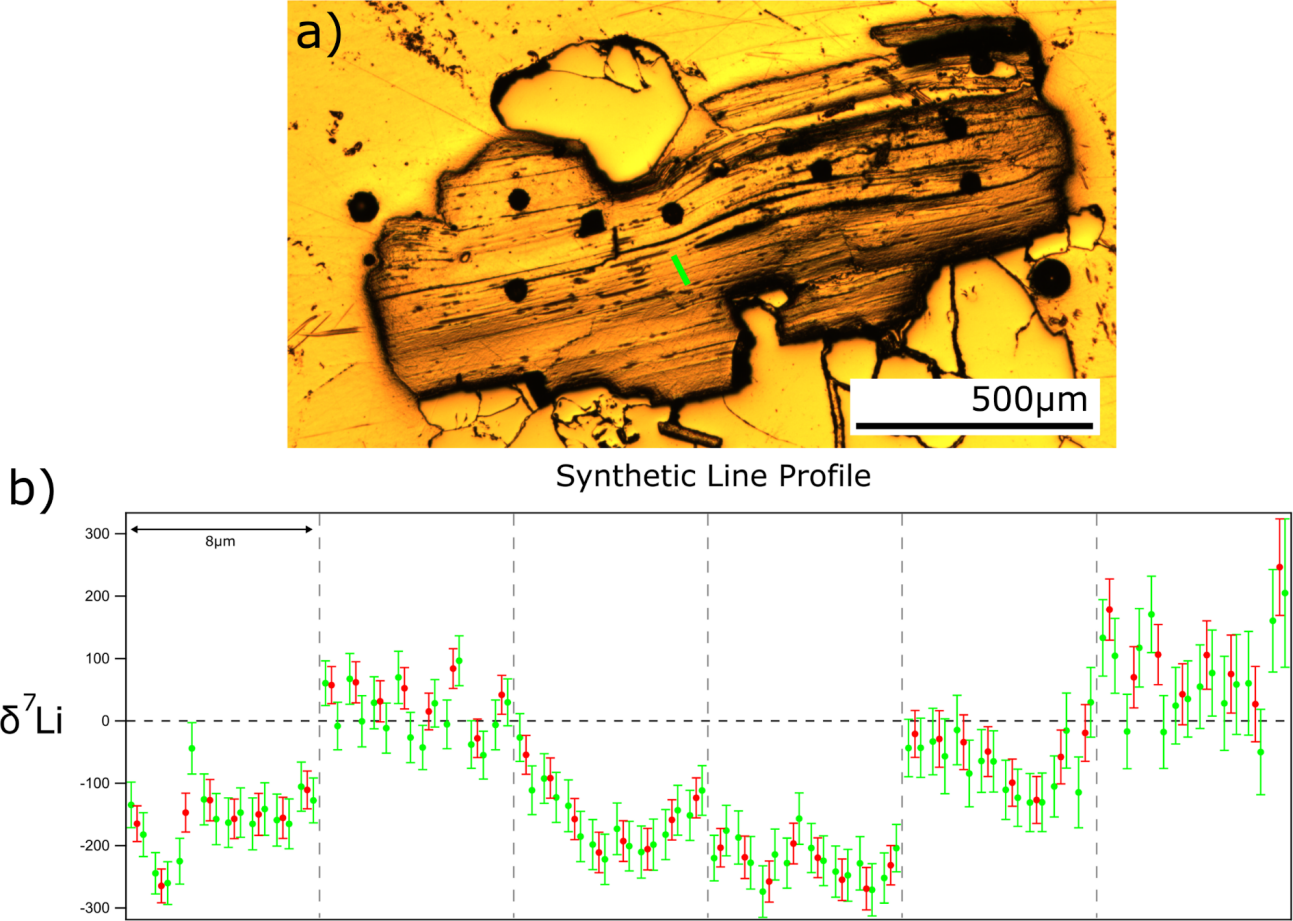
a) Examples of deformed cleavage planes in a Li-rich biotite mica shown parallel to the *c*-axis and b) δ^7^Li values for vertical strips taken perpendicular to the scanning direction of the beam, along the green line in a). Each vertical grey line represents two microns of space left between each image, whilst red values are calculated using 1/8th width strips of the original maps and green values are calculated using 1/16th strips. Values are normalised to the average ratio across all measurements of the sample, rather than to an external standard.

Lithium isotope line profiles have also been measured, taking sequential maps perpendicular to deformed cleavage planes found in a sample of Li-rich biotite mica. These deformed cleavage planes are shown perpendicular to the *c*-axis of the biotite in Figure 10a. Again, δ^7^Li values are calculated relative to the average across all measurements, showing variation from the average values, rather than true isotopic variation. Figure 10b shows the isotopic variations across the sample, calculated using the asymmetric regridding method outlined above. The effect of calculating values for strips of different widths can be seen, larger variations are smoothed out by summing over strips with larger widths. This demonstrates the effect of increased count rate, with smaller internal error for each point, for all points except two. The values calculated for wider strips lie within the error of the values for two strips of half width. The external error of both datasets is approximately constant at ca. ±127‰, which is too low to explain the entire variation through instrument effects alone, leaving true geological variation as a possible cause.

## Conclusion

The helium ion microscope provides an imaging tool with extreme spatial resolution using secondary electron imaging. With the addition of secondary ion mass spectrometry capabilities at the highest resolution, the HIM–SIMS is now set to fill a critical length-scale gap in the field of microanalysis, with resolutions second only to the atom probe, but with field of views of the order of micrometres, allowing for high resolution over a relatively large sample area. The HIM–SIMS is therefore a useful tool for a wide range of geological applications. The critical capability of sensitivity to light elements makes it an important tool for economic geology focused on a low-carbon future [19]. The experimentally measured sensitivity on the ppm level for Li is more than enough for the analysis of a wide range of economically important Li-bearing minerals. The spatial resolution over an area of micrometres yields exciting prospects in the field of planetary materials, where micrometre-sized or smaller inclusions hold important information about both the early solar system and the nature of extrasolar conditions [10].
